# Ex Situ Study on the Co-Preparation of Pitch and Carbon Black from Petroleum Residue to Improve the Cost-Efficiency of the Pitch Synthesis Plant

**DOI:** 10.3390/ma16093592

**Published:** 2023-05-08

**Authors:** Ji-Hong Kim

**Affiliations:** C1 Gas & Carbon Convergent Research Center, Korea Research Institute of Chemical Technology (KRICT), Daejeon 34114, Republic of Korea; pic10@krict.re.kr

**Keywords:** carbon black, pitch byproduct, pitch, pyrolyzed fuel oil, petroleum residue, pilot plant

## Abstract

This study aims to improve the economic efficiency of the pitch synthesis reaction on the pilot plant by optimizing the pitch synthesis reaction and utilization of the byproduct. The pitch was synthesized using a 150 L pilot plant with pyrolyzed fuel oil as a precursor. The pitch synthesis reaction is carried out through volatilization and polycondensation, which occur at 300 and 400 °C. Volatilization is terminated during heating; thus, additional soaking time is meaningless and reduces the process efficiency. Soaking time is a major variable when the synthesis temperature exceeds 400 °C. The byproduct is generated through volatilization; thus, its chemical characteristics are only influenced by the reaction temperature. The byproduct consists of various polycyclic aromatic hydrocarbons. The average molecular weight and yield of the byproduct increase with the reaction temperature. Carbon black was synthesized using chemical vapor deposition from the byproduct. The particle size of carbon black was controlled by the used precursor (byproduct), and the electrical conductivity of prepared carbon black has a maximum of 58.0 S/cm. Therefore, carbon black, which is synthesized from the byproduct of pitch synthesis, is expected to be used as a precursor for conductive material used in lithium-ion batteries or supercapacitors.

## 1. Introduction

Demand for carbon materials such as carbon fiber, synthetic graphite, and activated carbon has increased with the development of industries and technology. These carbon materials are manufactured using pitch, which is formable to various structures due to its thermoplasticity properties [1,2,3,4]. Polycyclic aromatic hydrocarbons (PAHs) such as coal tar and petroleum residue can be used as precursors for pitch. Regarding the preparation method for pitch, a simple thermal reaction, halogenation (using F, Cl, Br), and catalytic reaction (using ferrocene) were reported [5,6,7,8,9,10,11]. However, carbon materials generally require high purity, and removing the heteroatoms in pitch is hard; thus, the thermal reaction is usually used in the relevant industry [12,13,14,15].

Pitch is synthesized by volatilizing low-molecular-weight PAHs and polycondensation, which involves dehydrogenation and dealkylation [16,17,18]. The polycondensation reaction is required above 400 °C in the simple thermal reaction, and an anisotropic phase (called mesophase) is formed as a result. This means that the chemical species, which have a boiling point under 400 °C, were removed by volatilization during the heating process. In general, although there are differences depending on the average molecular weight of the precursors used or reaction temperature, the yield of pitch synthesis is hard to extend beyond 40 wt.% [18,19,20]. This low yield is a hurdle for the commercialization of pitch-based carbon materials in terms of energy cost and disposal of volatiles.

These volatiles can be used as precursors for carbon blacks. Carbon black is synthesized via pyrolysis or the combustion of hydrocarbons in the gas phase. Specifically, PAHs are generated during the pyrolysis of hydrocarbons and work as carbon black nuclei [21,22]. In the case of volatiles, there are abundant low molecular-weight PAHs which are easily vaporized; thus, the volatiles have the advantage of precursors for carbon blacks. Moreover, the demand for carbon black recently increased for conducting materials in lithium-ion batteries; thus, volatile-based carbon black also has the advantage from an economic perspective.

This study suggests the utilization method of these volatiles for commercializing pitch pilot plants. Concretely, the applicability of the volatiles to precursors for carbon black is discussed [23,24,25,26]. The volatiles were prepared using a 150 L scale pilot plant as a byproduct of pitch synthesis. Carbon black was synthesized using the volatiles as a precursor in the chemical vapor deposition system. In particular, when considering various pitch synthesis conditions, the effect of the characteristics of volatiles is first observed according to the reaction temperature, then the effect of the chemical structure of volatiles on the size of carbon black is studied. This study is expected to provide an understanding of pitch synthesis behavior on the pilot-scale reactor and enhance its economic efficiency.

## 2. Materials and Methods

### 2.1. Materials

Pyrolyzed fuel oil (PFO; LG chem, Seoul, Republic of Korea), a byproduct of the naphtha cracking center of the petrochemical process, was used as a precursor to synthesize the pitch and carbon black without purification. The characteristics of the PFO used are presented in Table 1.

### 2.2. Pitch Synthesis

**Figure 1:**
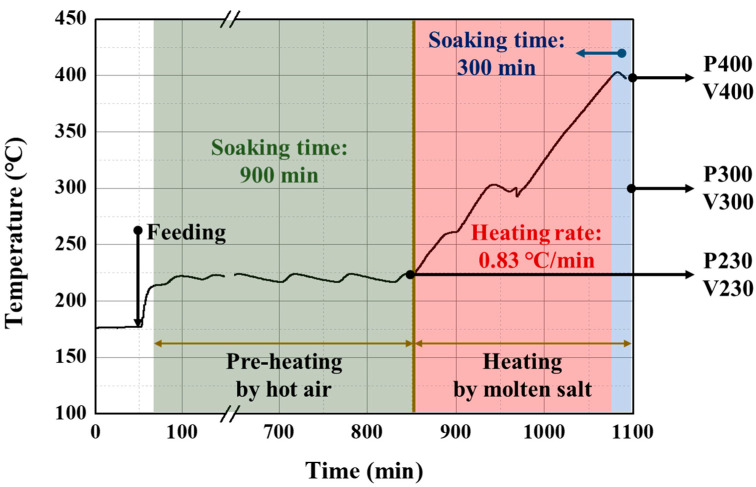
Temperature profile of 150 L pilot plant used for pitch synthesis; (green) pre-heating, (red) heating, and (blue) soaking range.

Pitch samples were prepared using a 150 L scale pilot plant, as shown in Figure S1. Pipelines of the pilot plant were heated to 200 °C before the injection of PFO in the reactor. After heating pipelines, 80 kg of PFO was transferred from the feed tank to the reactor. The temperature of the reactor was indirectly controlled by heated molten salt. Molten salt solidifies below 220 °C, so the reactor was pre-heated using hot air at 230 °C for 900 min. After pre-heating, the reactor was heated to the target temperature by a heating rate of 0.83 °C/min, and the reaction was soaked for 300 min after reaching the target temperature. The prepared pitch was transferred to a sampling tank in its liquid state and sampled after natural cooling to the solid state. During pitch synthesis, generated volatiles were liquified using a condenser kept at 30 °C, and the liquid volatiles were collected in the oil tank. Uncondensed volatile gases were removed through a 2-step process: a wet scrubber, and adsorption to activated carbon. The prepared sample was named as the target temperature, and at this time, P and V were attached to pitch and volatile samples, respectively. For example, prepared pitch and volatiles at 300 °C were named P300 and V300. The P230 and V230 sample was taken after pre-heating. The temperature profiles and simplified process procedure of the pilot plant are expressed in Figure 1.

### 2.3. Characterization of PFO and Volatile Samples

The chemical compositions of PFO were analyzed by simulated distillation gas chromatography (GC-SIMDIS). A total of 0.2 g of PFO was mixed with 6.8 g of carbon disulfide (Samchun Chemical Co., Ltd., Seoul, Republic of Korea), and the analysis proceeded following ASTM D 7169. Thermogravimetric analysis (TGA; Thermo Plus EVO II TG8120, Rigaku, Tokyo, Japan) was used to predict the thermal reaction behavior of PFO, and 5 mg of PFO was heated at 5 °C/min to 900 °C under inert conditions. The chemical structure of prepared volatile samples was estimated by GC-SIMDIS (the same condition as PFO) and gas chromatography–mass spectrometry (GC-MS).

### 2.4. Carbon Black Preparation

**Figure 2:**
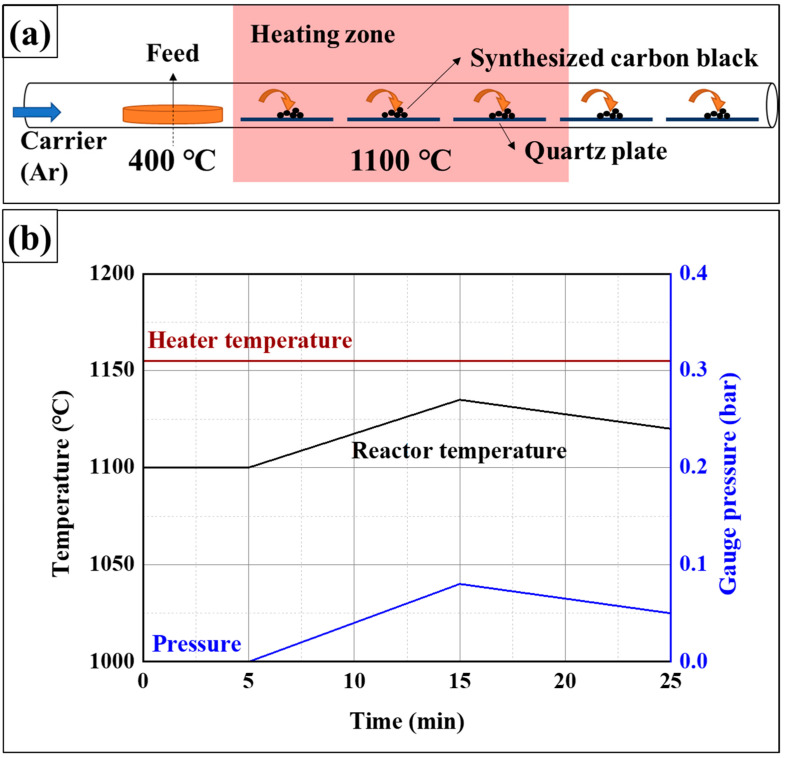
Process for the preparation of carbon black, (**a**) schematic diagram of chemical vapor deposition, and (**b**) temperature and pressure profiles.

A chemical vapor deposition (CVD) process was employed to prepare carbon black from the volatiles. An electric furnace was set to 1100 °C, and a quartz boat containing the volatiles was placed in a position maintained at 400 °C by the temperature gradient. The process schematic for carbon black preparation is expressed in Figure 2a. Figure 2b shows a temperature and gauge pressure profile during the CVD process. Before supplying the volatiles, the maintenance of reaction temperature was checked for 5 min. The reaction rapidly proceeded as soon as the volatiles were supplied, and it showed that the reactor’s temperature increased as a result of the heat transfer to the thermocouple performed by the reactant. The gauge pressure also slightly increased due to the blockage caused by the rapidly formed carbon black. The increase in temperature and gauge pressure were stopped after 10 min with the termination of the reaction. The prepared samples were named CBx; x is the generated temperature of volatile samples, which was used as the precursor for carbon black.

### 2.5. Characterization of Carbon Black

Field-emission scanning electron microscopy (FE-SEM) micrographs taken with the acceleration voltage of an electron beam of 10 kV and magnification of 3000 were used to observe the size and shape of the prepared carbon black. The electrical conductivity of carbon black powder was investigated by the HPRM-FA2 (HANTECH, Gunpo, Republic of Korea) Powder Resistivity Measurement System. Prepared carbon black was pressed in a 22 mm diameter cylindrical sus mold. The electrical conductivity was measured via a 4-probe method under a pressure range of 400–2000 kgf.

## 3. Results and Discussion

### 3.1. Preparation of Pitch and Volatiles on Pilot Plant

#### 3.1.1. Predict of Pitch Synthesis Reaction of PFO

**Figure 3:**
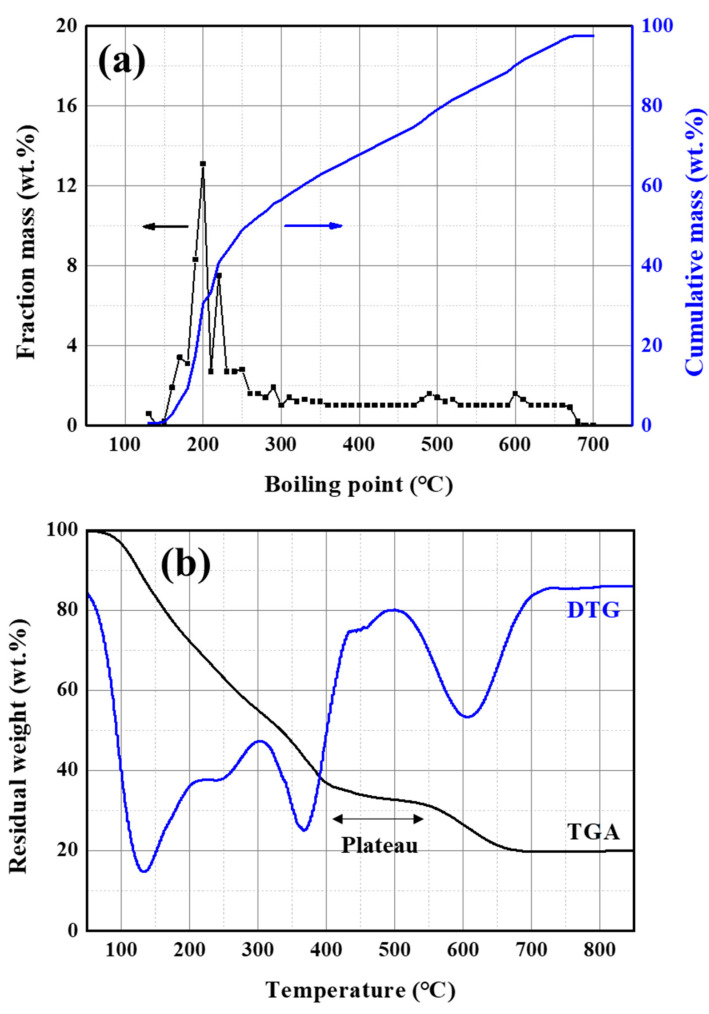
(**a**) GC-SIMDIS curves of PFO, and (**b**) thermogravimetric curves of PFO.

To predict the pitch synthesis reaction of PFO, GC-SIMDIS, and TGA were used, as shown in Figure 3. In Figure 3a, GC-SIMDIS was carried out to investigate the chemical composition based on the boiling point of the feedstocks. In GC-SIMDIS, 2 peaks near 200 °C and broad distribution from 300 to 700 °C can be observed. In the SARA analysis in Table 1, a saturated fraction was not detected in PFO. Therefore, the peaks near 200 °C seem to be naphthalene (BP: 218 °C) with aliphatic functional groups. In Figure 3b, the derivative thermogravimetric, calculated from TG, shows 4 peaks at 130, 200, 370, and 600 °C. In TGA, the weight change by complex reactions, including volatilization and polycondensation, is observed. Peaks of 130 and 200 °C are considered the same peak, near 200 °C, in GC-SIMDIS. The inert gas is injected at 100 cc/min in TGA conditions. Therefore, volatilization can be easily generated by the dried inert gas, which acts like a carrier gas.

When overlapping the cumulative mass of GC-SIMDIS and the residual weight of TGA, they show a different trend at 400 °C (see Figure S2). In TGA, the plateau at 400 to 500 °C occurred. In general, PAHs express reactivity to polycondensation above 400 °C. Polycondensation of PAHs is initiated by dehydrogenation or dealkylation, and the weight is decreased by the release of generated hydrogen or methane [16,17,18]. Figure S3 shows the elemental contents of volatiles and pitch prepared by a 5 L lab-scale reactor. The H/C atomic ratio of the prepared pitch dramatically decreased above 400 °C in Figure S3b. However, the H/C ratio of the volatiles in Figure S3a is constant throughout the whole reaction temperature because the gas product generated by dehydrogenation and alkylation cannot be condensed under 30 °C. Moreover, polycondensation causes the phase change from iso-tropic to anisotropic (called mesophase) because the polymerized PAHs are stacked via pi–pi interaction; the mesophase is only observed when synthesized at 420 °C, as shown in Figure S4 [5,16].

In summary, the pitch synthesis of PFO is proceeded by volatilization and polycondensation. Volatilization continuously occurs under 400 °C, and the volatiles are maximumly generated at 200 °C by massive amounts of naphthalene (almost 40 wt.%). Polycondensation occurs at temperatures above 400 °C, and changes in pitch characteristics rapidly progress, such as polymerization and mesophase formation; however, the characteristics of volatiles only show little changes (see Figure S5).

#### 3.1.2. Preparation of Pitch and Volatiles in the Pilot Plant

The conditions for pitch synthesis were set to 230, 300, and 400 °C to control the characteristics of volatiles based on the number of aromatics. The general characteristics of the prepared pitch and volatiles are listed in Table 2.

**Figure 4:**
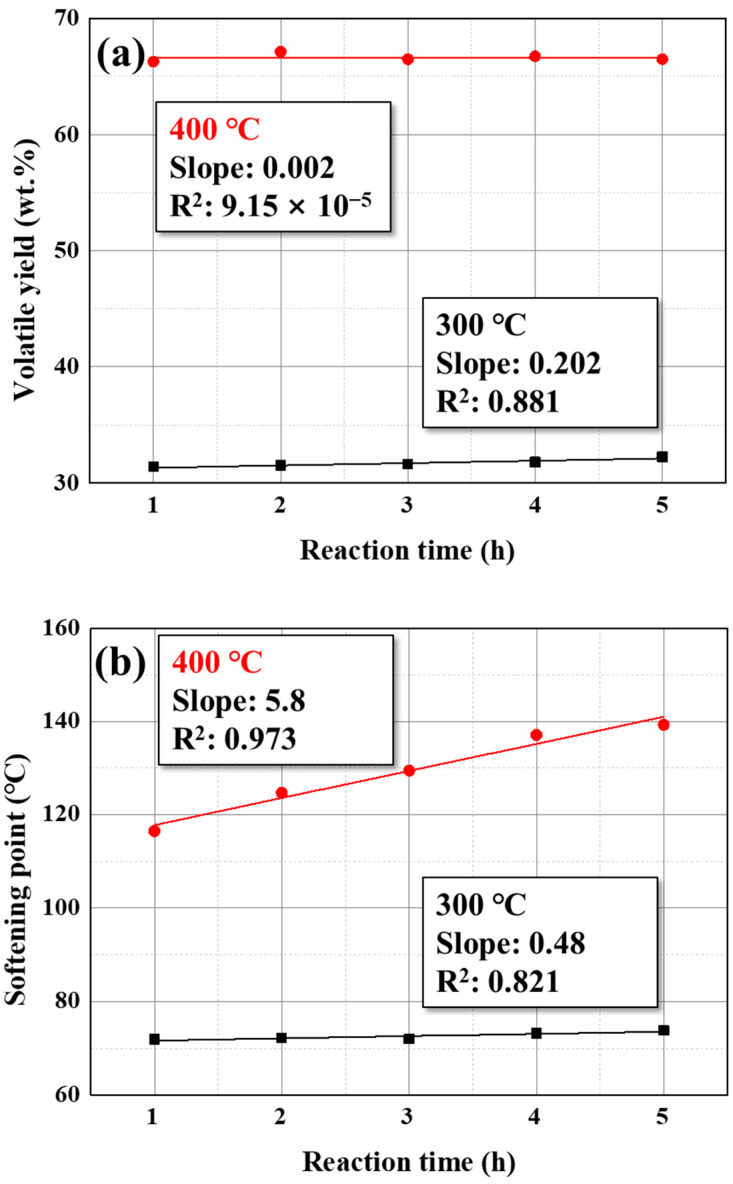
(**a**) Prepared volatile yield and (**b**) softening point of pitch, according to reaction times.

Regarding yield, pitch decreased with an increasing reaction temperature by volatilizing the low-molecular-weight composition. Understandably, the volatiles increased as much as the loss of pitch yield. This resulted in gas products, such as hydrogen and methane, due to dehydrogenation and dealkylation during polycondensation, and they were under 1 wt.% under 300 °C; however, the gas yield rapidly increased at 400 °C to 2.42 wt.%. This means polycondensation proceeds above 400 °C in the pilot plant, as is the case with the 5 L lab-scale reactor. The change in volatile yield and softening point for 5 h was investigated to consider the pitch synthesis behavior according to the reaction temperature, as shown in Figure 4.

As shown in Figure 4a, during 5 h of reaction, the volatile yield was almost constantly maintained at 32.26 and 66.48 wt.% at 300 and 400 °C, respectively. This means that volatilization rapidly occurred during heating to the target temperature. The slope of the fitting curve is higher at 300 °C than at 400 °C because the higher reaction temperature has the advantage in volatilization. That is, the unvolatilized composition relatively increases higher in lower reaction temperatures. For the same reason, the correlation coefficient R^2^ at 400 °C has a low value, 9.15 × 10^−5^. The volatile yield is virtually not changed after reaching the target temperature. Therefore, the R^2^ is very sensitive to minor errors in volatile yield.

In Figure 4b, the reaction temperature for polycondensation can be observed by the changes in the softening point. The softening point of the pitch prepared at 300 °C was consistently maintained at 73.8 °C. Comparing the tendency of volatile yield and softening point at 300 °C, the pitch synthesis reaction was majorly influenced by volatilization under 300 °C. At higher temperatures, more volatilization occurs. Therefore, controlling the temperature is more effective in controlling the softening point of pitch. Moreover, the reaction times are meaningless because volatilization almost terminated during the heating process, reducing the process efficiency.

In contrast, the softening point of the pitch prepared at 400 °C continuously increases with the reaction time. As mentioned earlier, volatilization is almost terminated during the heating process; thus, this change in the softening point affects polymerization. Polycondensation is influenced by both reaction temperature and time. Therefore, these factors must be set carefully to control the characteristics of the pitch.

**Figure 5:**
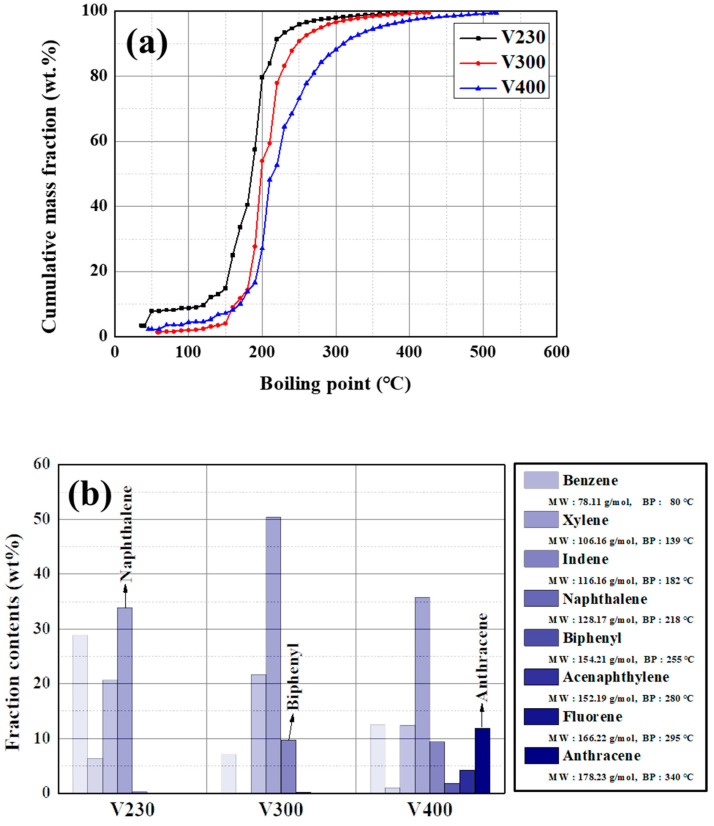
(**a**) GC-SIMDIS curves and (**b**) fraction contents calculated from GC-MS of prepared volatile samples.

To investigate the component of prepared volatiles, GC-SIMDIS and GS-MS were used, as shown in Figure 5. As mentioned in Section 3.1, prepared volatiles mainly comprise naphthalene with functional groups. Thus, the cumulative mass fraction rapidly increased at 200 °C in all volatile samples shown in Figure 5a. Based on the curve of V300, V230 and V400 are located to the left and right, respectively. From these results, it is predicted that the volatiles can be easily controlled by the reaction temperature. The composition of volatiles is listed in Figure 5b. In the GC-MS, the volatiles have complex compositions; thus, the fraction contents were summed based on their main chemicals, benzene, xylene, indene, naphthalene, biphenyl, acenaphthylene, fluorene, and anthracene. For example, naphthalene, 2-methyl-naphthalene, 1,6-dimethyl-naphthalene, and 2-ethyl-naphthalene were summed into the naphthalene fraction. The GC-MS result also shows the same tendency as GC-SIMDIS; naphthalene has a maximum fraction in whole volatile samples. Moreover, the heaviest chemicals in V230, V300, and V400 are naphthalene, biphenyl, and anthracene, respectively; thus, the average molecular weight and size increase with the reaction temperature.

Additionally, the sulfur content, nitrogen content, and total calorific value of volatiles were investigated to consider their use as fuel, as listed in Table 2. The volatiles commonly have 42 MJ/kg of total calorific value, and this total calorific value is similar to kerosene. Moreover, the volatiles prepared from PFO have very low sulfur and nitrogen, below 0.03 and 0.09 wt.%, respectively. Therefore, it is expected that the volatiles can potentially be applied as fuel oil.

### 3.2. Preparation of Carbon Black via Chemical Vapor Deposition

This chapter considers the effect of the chemical structure of precursors (volatiles) on the synthesis of carbon black. As shown in Figure 2, the synthesis temperature for carbon black was set to 1100 °C experimentally. (Under 1000 °C, the efficiency of chemical vapor deposition and the yield of carbon black was low.) The structure of carbon black shows an aggregation of sphere particles, and the sphere particle is called the primary particle. The size of the primary particle was influenced by the position in the quartz tube of products and used precursors, as shown in Figure S6. The size of the primary particle rapidly decreased with an increasing distance from the heating zone because the flight time of generated particles in carbon black can be increased with smaller sizes [24]. Additional analysis progressed using the carbon blacks generated in the heating zone to consider the effect of the chemical structure of volatiles on prepared carbon blacks.

**Figure 6:**
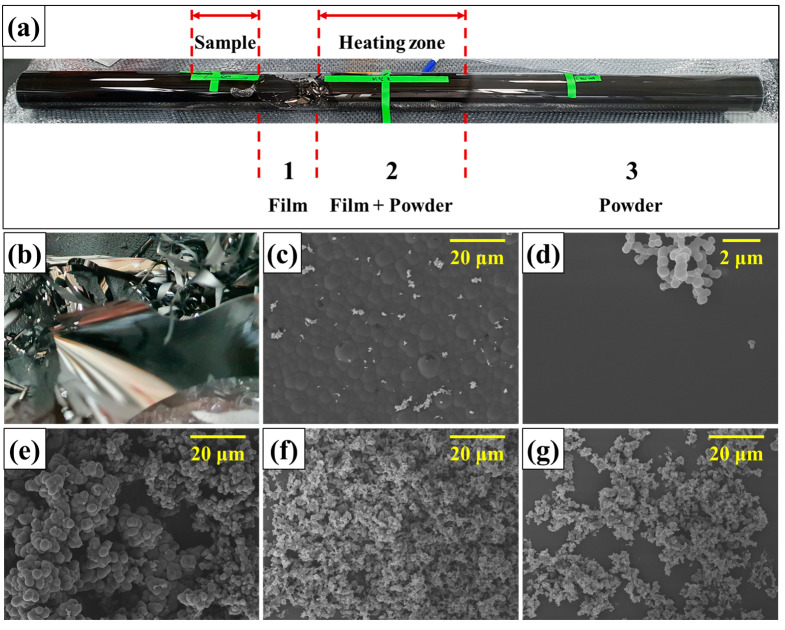
Prepared samples by CVD process, (**a**) full picture of quartz tube, (**b**) film-type sample, (**c**) FE-SEM image for external part of prepared film, (**d**) FE-SEM image for internal part of prepared film, (**e**) FE-SEM image for CB400, (**f**) FE-SEM image for CB300, and (**g**) FE-SEM image for CB230.

The FE-SEM images of the prepared samples are shown in Figure 6. In Figure 6a, two kinds of products were formed after CVD. One is a film-shaped carbon (Figure 6b), and the other is carbon black powder (Figure 6e–g). In the film-type sample, the external part did not exhibit a particular shape, showing a smooth surface in Figure 6c. However, the internal part showed an aggregation of spherical carbon bodies, as shown in Figure 6d. The film-type samples were only observed near the heating zone, and it is considered that the film is formed by highly aggregating carbon blacks by continuous thermal reactions. Additional study is required to discover the formation mechanism and characteristics of the film.

The primary particle size of prepared carbon black increased with the average size of composed chemicals in volatiles, and the particle size is 0.7, 1.5, and 3.2 μm in CB230, CB300, and CB400. In the CVD process, the carbon material is synthesized by the packing of vapors. In the volatiles, aromatics are more thermally stable than functional groups. Therefore, the cracking of functional groups first occurs, then the remaining PAHs are grown to carbon black. Due to this, the particle size of carbon black can be increased with the average size of composed chemicals in volatiles [26]. Moreover, decomposed functional groups can be deposited on the defect of carbon black; thus, the electrical and thermal conductivity can be improved by chemical packing [27].

**Figure 7:**
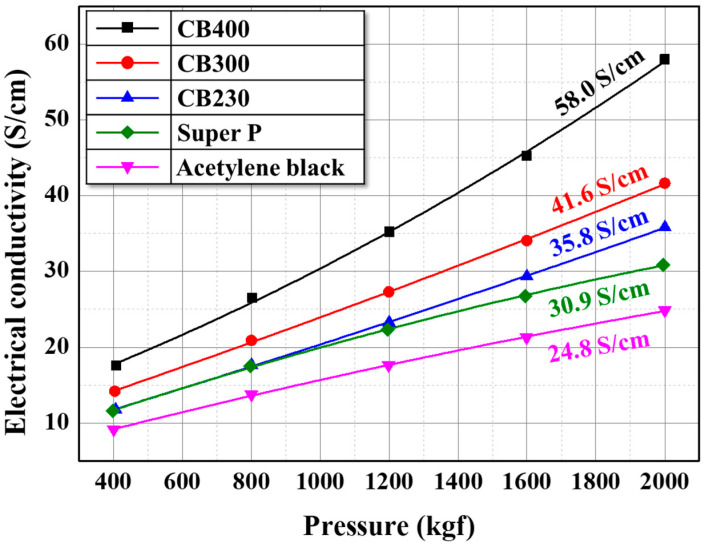
Electrical conductivity of prepared samples and commercial carbon black.

The applicability of the prepared carbon black as a conductive material was investigated by evaluating the electrical conductivity, as shown in Figure 7. The electrical conductivity of prepared carbon black is 35.8, 41.6, and 58.0 S/cm in CB230, CB300, and CB400, respectively. In this study, the electrical conductivity was measured in the packing state of carbon black powder; thus, the packing properties, such as particle size distribution, also influence it. Therefore, it is hard to find meaningful consideration in comparing CB230, CB300, and CB400. However, the prepared carbon black has higher electrical conductivity than the commercial conductive material, Super P (30.9 S/cm) and acetylene black (24.8 S/cm). This result is considered the chemical packing effect, as mentioned earlier [27]. Therefore, the volatiles can be used as a precursor for conductive material for lithium-ion batteries or electrical double-layer capacitors in size control and electrical conductivity.

## 4. Conclusions

This study considered the analysis of a pitch synthesis byproduct (volatiles) and the utilization of conductive carbon black to improve the economic efficiency of a 150 L pitch synthesis pilot plant.

Pitch synthesis is proceeded by volatilization and polycondensation. When PFO is used as a precursor, volatilization and polycondensation mainly proceed at 300 and 400 °C, respectively. Volatilization is terminated during the heating process to the target temperature. Thus, the reaction temperature is a key variable affecting the characteristics of the pitch and volatiles. The soaking time is meaningless under a reaction temperature of 300 °C, and it reduces the cost efficiency of the process by increasing the energy cost. However, the reaction temperature and soaking time are major factors when pitch synthesis exceeds 400 °C. Therefore, the reaction temperature and soaking times must be suitable to consider the target properties.

The average molecular weight of volatiles and their yield increase with increasing reaction temperatures. However, above 400 °C, this tendency disappeared because polycondensation mainly occurred. During polycondensation, hydrogen and methane are mainly generated by dehydrogenation and dealkylation. Since these components are not condensed in the condenser set at 30 °C, they cannot affect the change in volatile components.

Carbon black, which is synthesized from volatiles in CVD, is influenced by the characteristics of the used precursor, reaction temperature, and flow rate of purging gas. The primary particle size of carbon black increased with the generating temperature of volatiles because the average molecular weight of volatiles increases with the reaction temperature. Prepared carbon black from volatiles results in higher conductivity than the commercial conducting materials Super P and acetylene black. It is considered that the small molecules generated by the cracking of functional groups cause the chemical packing on carbon black, so the defect of carbon black is reduced.

Additionally, it is expected that nanomaterials such as carbon nanotubes and graphene are also synthesized from volatiles by CVD with Cu-, Fe-, and Ni-based catalysts [27,28,29,30,31]. Conclusively, this study suggests the combination of a pitch synthesis reactor and CVD to manufacture pitch, carbon nanomaterials, and carbon black simultaneously to improve the economic efficiency of pitch commercialization.

## Figures and Tables

**Table 1:** Properties of the precursor, pyrolyzed fuel oil.

Properties		Unit	Value
Density		g/cm^3^	1.064
Viscosity		cP	500
SARA analysis	Saturated	%	-
	Aromatics	%	93.91
	Resin	%	5.77
	Asphaltenes	%	0.32
Ash ^1^		wt.%	0.25
Carbon value ^2^		wt.%	8.97
Elemental analysis	Carbon	%	92.41
	Hydrogen	%	7.35
	Nitrogen	%	0.02
	Sulfur	%	0.04
	H/C atomic ratio ^3^	-	0.95

^1^ Ash was estimated by residual weight at 800 °C of TGA in air atmospheric conditions. ^2^ Carbon value was estimated by residual weight at 900 °C of TGA in inert conditions. ^3^ H/C atomic ratio = $\frac{Hydrogen\;content\;(\%)}{Carbon\;content\;\left(\%\right)/12}$.

**Table 2:** Properties of the prepared pitch and volatile.

**Temp** **(°C)**	**Time** **(min)**	**Pitch Properties**		**Volatile (Liquid) Properties**				**Gas Yield** **(Cal ^3^. wt.%)**
		Yield (wt.%)	SP ^1^ (℃)	Yield (wt.%)	Sulfur (wt.%)	Nitrogen (wt.%)	TCV ^2^ (MJ/Kg)	
230	-	71.2 (Cal ^4^)	Semi-solid	28.8 ± 0.05	0.01	0.09	42.51	-
300	300	66.75 ± 0.13	73.8 ± 2.1	32.26 ± 0.87	0.03	0.07	41.64	0.99
400	300	31.10 ± 0.08	139.3 ± 4.3	66.48 ± 1.02	0.02	0.09	42.06	2.42

^1^ SP: softening point, ^2^ TCV: total calorific value, ^3^ gas yield (%) = 100 (%) − pitch yield (%) − volatile yield (%), ^4^ pitch yield at 230 °C (%) = 100 (%) − volatile yield (%).

## Data Availability

Data are contained within the article.

## Supplementary Materials

The following supporting information can be downloaded at: https://www.mdpi.com/article/10.3390/ma16093592/s1, Figure S1: P & ID diagram and process schematics of 150 L scale pilot plant for pitch synthesis; Figure S2: Comparing the cumulative mass of GC-SIMDIS and residual weight of TGA; Figure S3: Elemental contents of prepared pitch and volatile, according to the reaction temperature; Figure S4: Image of polarized optical microscopy of the prepared pitch at 360–420 °C; Figure S5: Fraction contents of volatiles calculated from GC-MS at 360–420 °C; Figure S6: FE-SEM images of prepared carbon black according to the used precursors and position of the reaction tube.
