# Supplementary material for: Ex Situ Study on the Co-Preparation of Pitch and Carbon Black from Petroleum Residue to Improve the Cost-Efficiency of the Pitch Synthesis Plant
Source: Materials (Basel). 2023 May 8;16(9):3592. doi: 10.3390/ma16093592 (PMC10180152; doi:10.3390/ma16093592)
Supplement: Supplementary file 1 [file materials-16-03592-s001.zip › materials-2345617-supplementary.pdf]

Supplementary Information

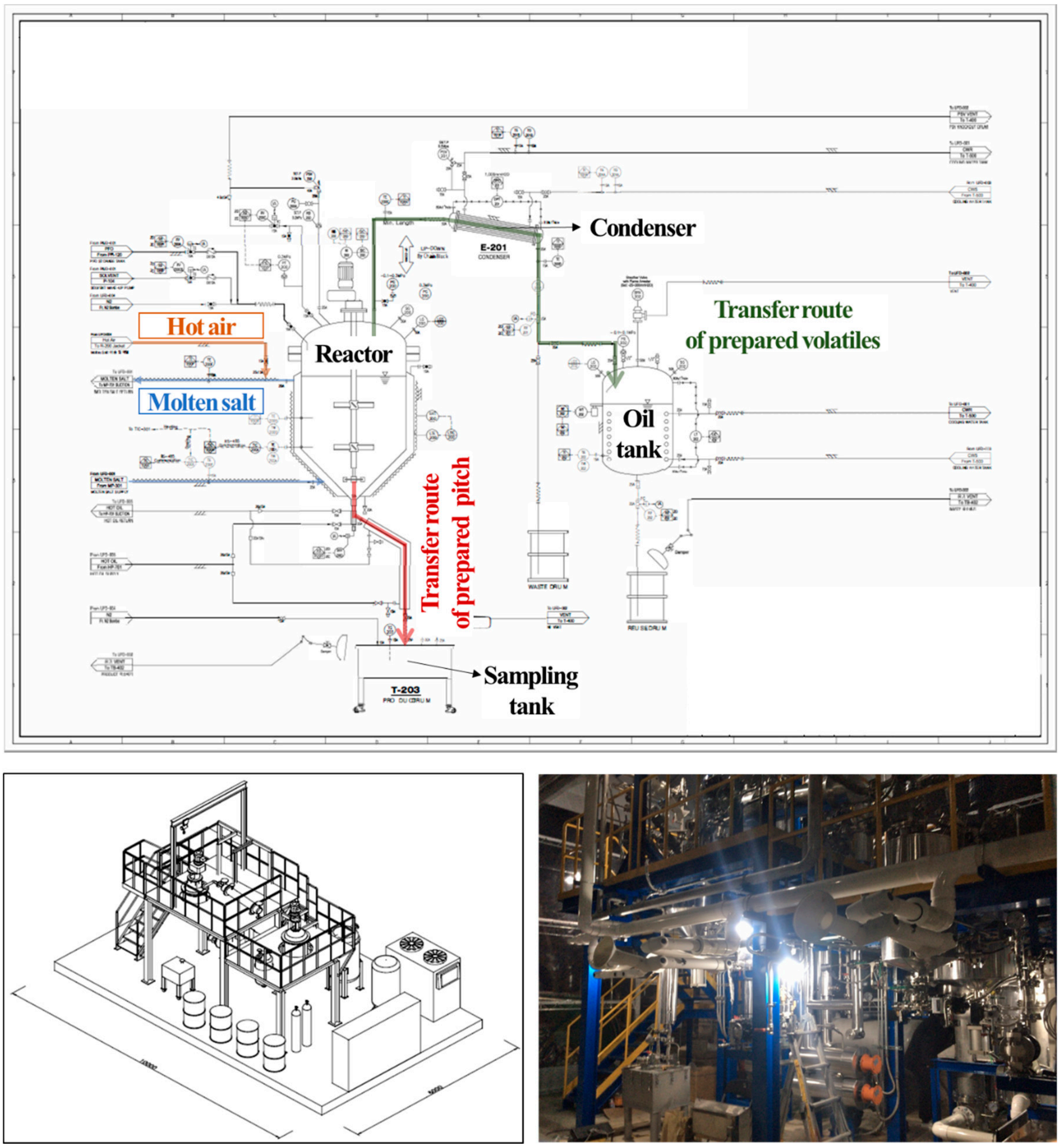

Figure S1. P&ID diagram and process schematics of 150L scale pilot plant for pitch synthesis.

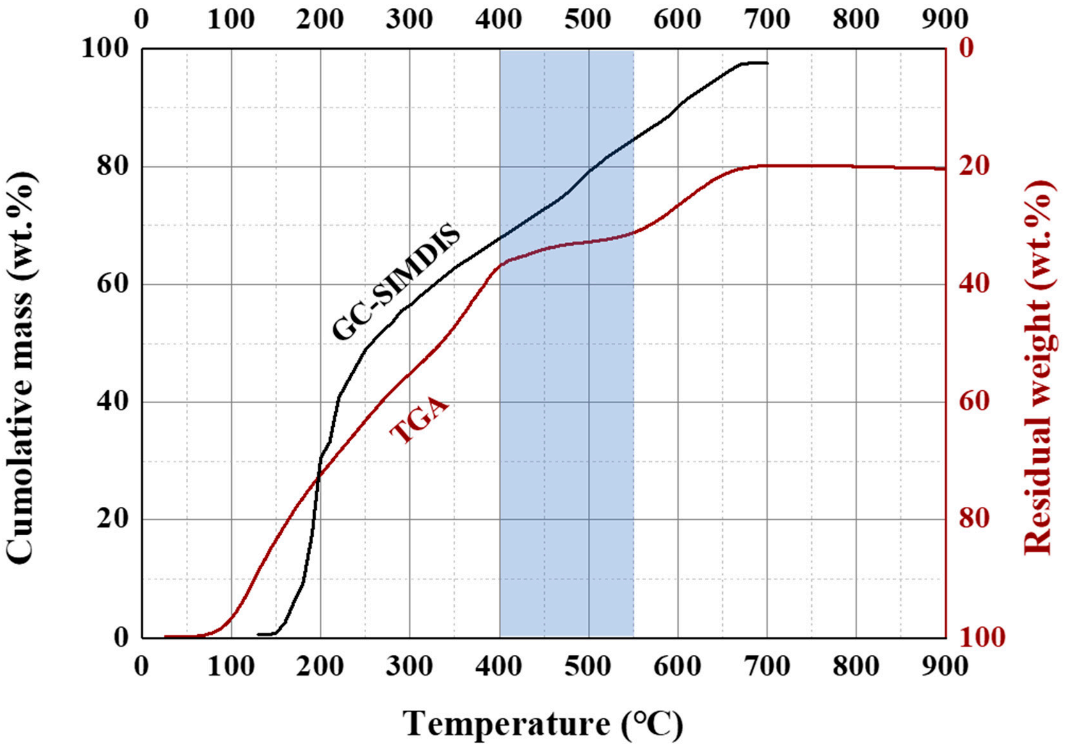

Figure S2. Comparing the cumulative mass of GC-SIMDIS and residual weight of TGA.

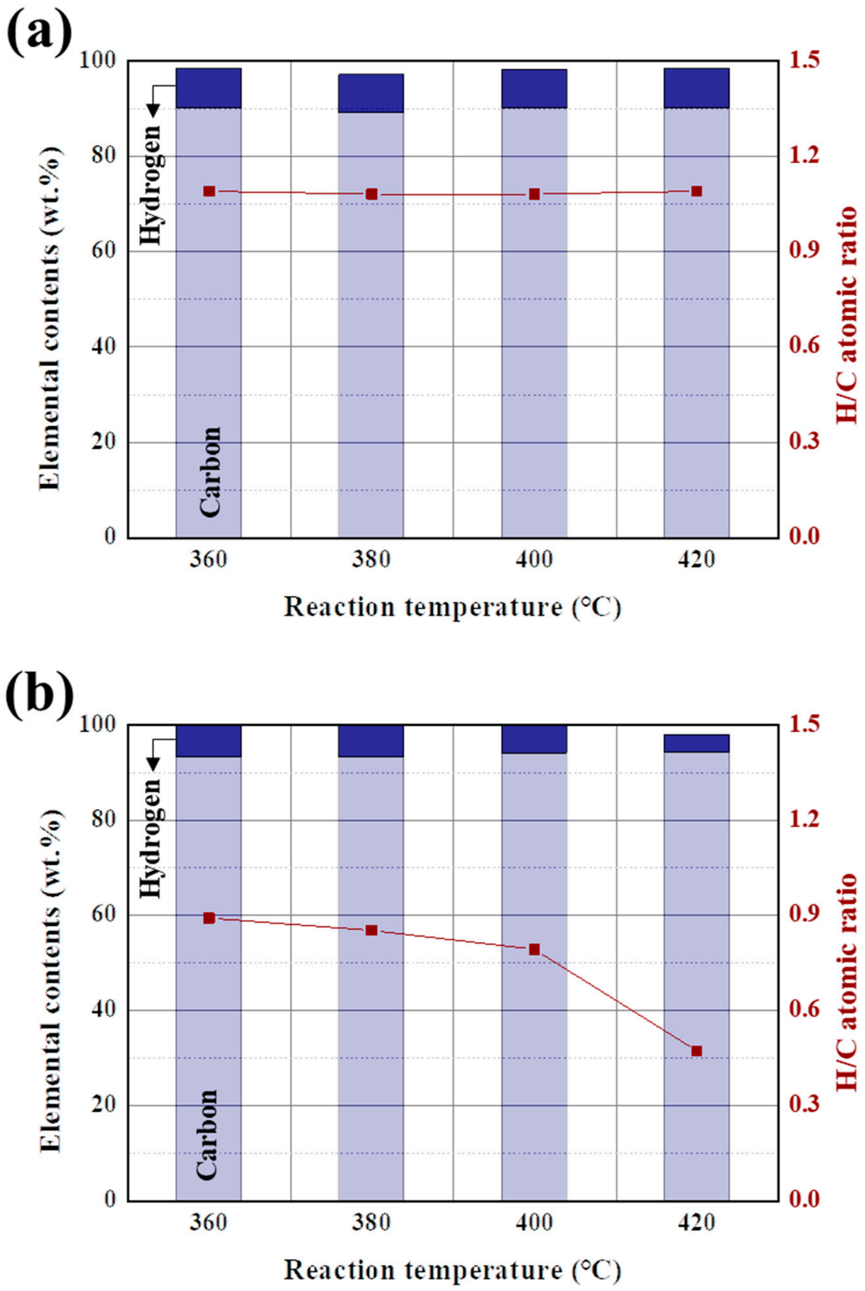

Figure S3. Elemental contents of (a) prepared pitch and (b) volatile, according to the reaction temperature.

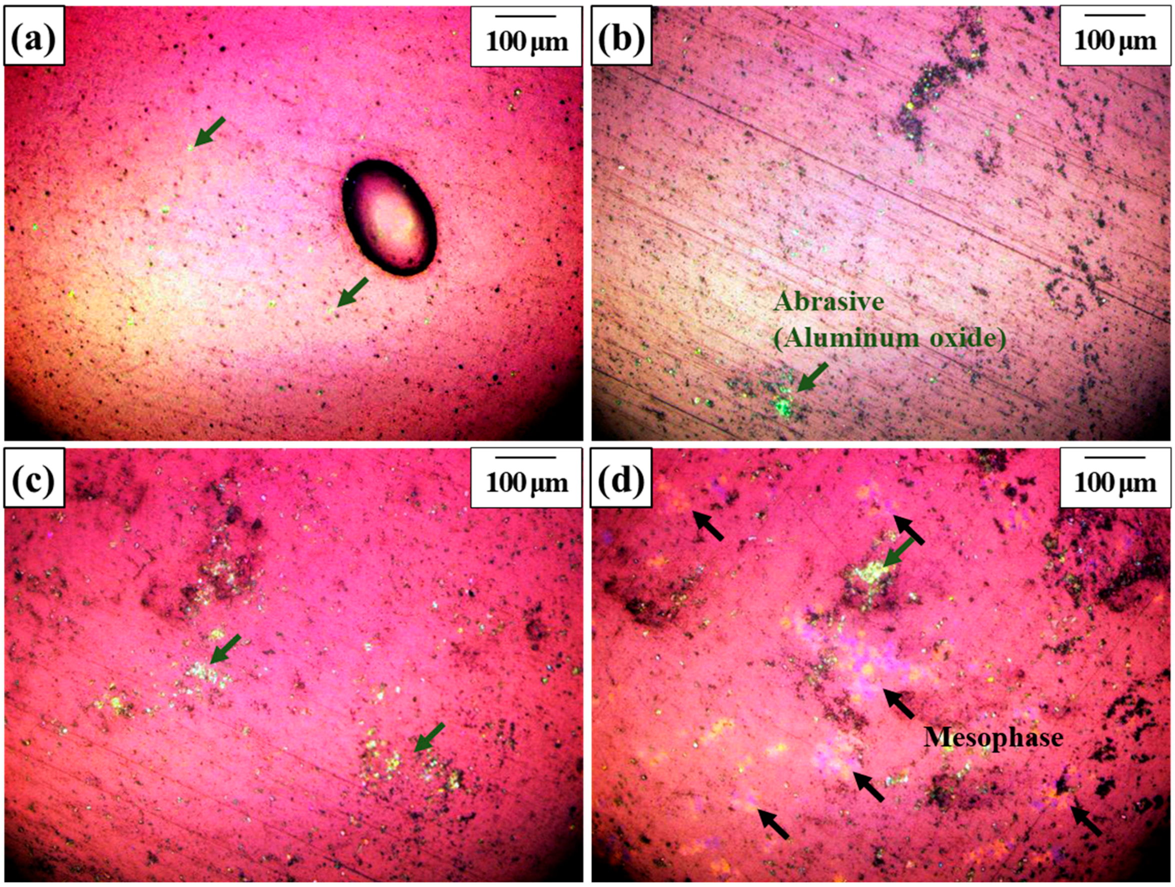

Figure S4. Image of polarized optical microscopy of the prepared pitch at (a) 360°C, (b) 380°C, (c) 400°C, and (d) 420°C.

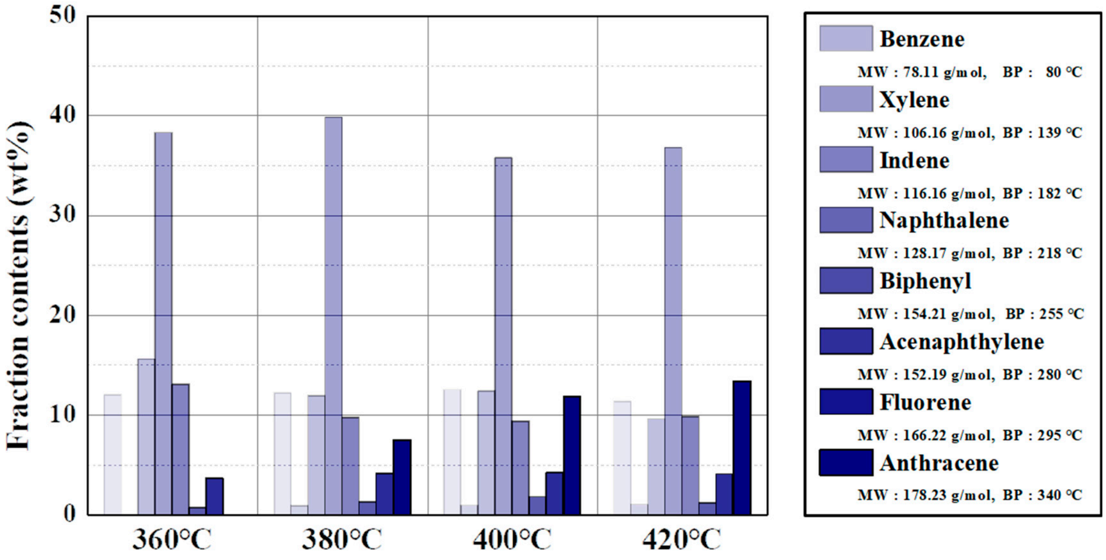

Figure S5. Fraction contents of volatiles calculated from GC-MS at 360-420 °C.

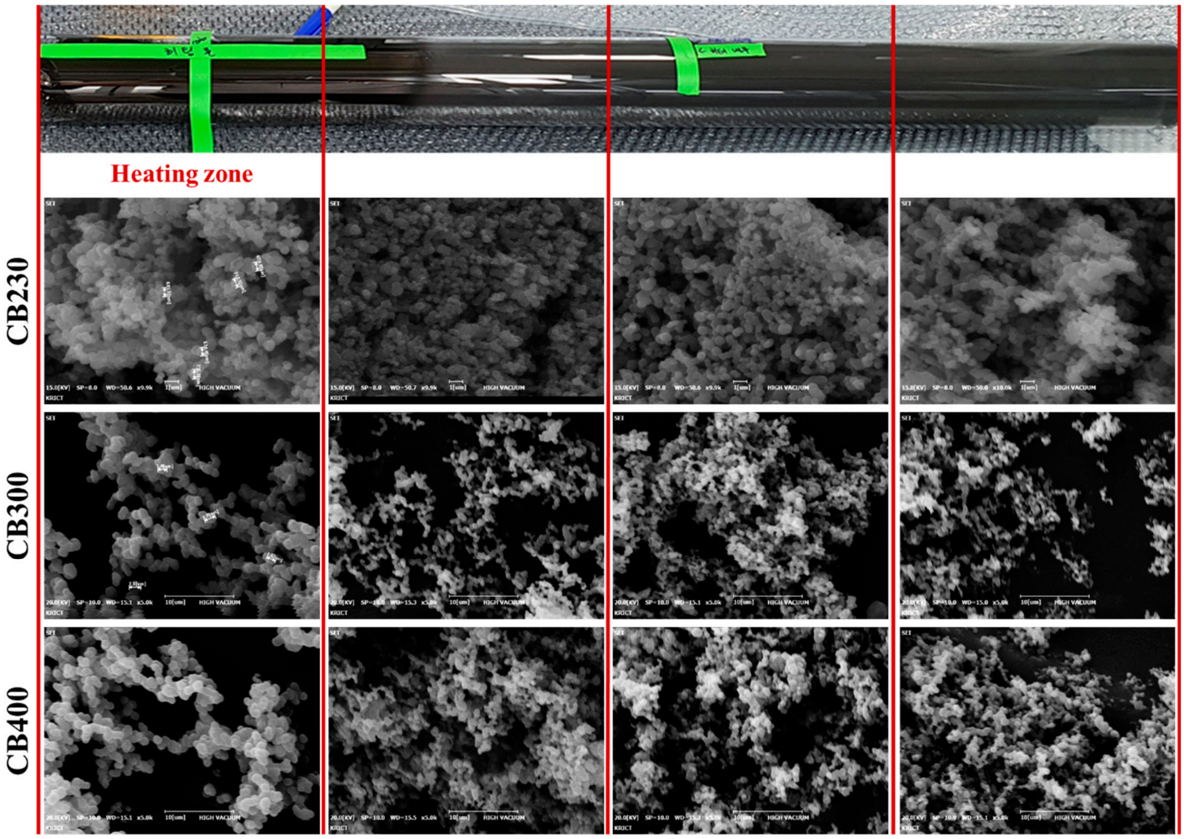

Figure S6. FE-SEM images of prepared carbon black according to the used precursors and position of the reaction tube.
